# Delivery of microRNA-302a-3p by APTES modified hydroxyapatite nanoparticles to promote osteogenic differentiation in vitro

**DOI:** 10.1038/s41405-023-00135-x

**Published:** 2023-02-22

**Authors:** Pirawish Limlawan, Laurine Marger, Stéphane Durual, Anjalee Vacharaksa

**Affiliations:** 1 Department of Microbiology, Faculty of Dentistry, Chulalongkorn University, Pathumwan, Bangkok, 10330 Thailand; 2 Research Unit on Oral Microbiology and Immunology, Faculty of Dentistry, Chulalongkorn University, Pathumwan, Bangkok, 10330 Thailand; 3 Biomaterials Laboratory, Division of Fixed Prosthodontics and Biomaterials, University Clinics of Dental Medicine, University of Geneva, 1 Rue Michel Servet, 1204 Geneva, Switzerland; 4 Master of Science Program in Geriatric Dentistry and Special Patients Care, Faculty of Dentistry, Chulalongkorn University, Pathumwan, Bangkok, 10330 Thailand

**Keywords:** Dental materials, Dental treatments

## Abstract

**Objective:**

To demonstrate the miRNA delivery by hydroxyapatite nanoparticles modified with APTES (HA-NPs-APTES) and promote osteogenic gene expression.

**Materials and methods:**

Osteosarcoma cells (HOS, MG-63) and primary human mandibular osteoblasts (HmOBs) were co-cultured with HA-NPs-APTES conjugated with miRNA-302a-3p. Resazurin reduction assay was performed to evaluate HA-NPs-APTES biocompatibility. Intracellular uptake was demonstrated by confocal fluorescent and scanning electron microscopy. The miRNA-302a-3p and its mRNA targets expression levels including COUP-TFII and other osteogenic genes were assessed by qPCR on day1 or day5 post-delivery. Calcium deposition induced by the osteogenic gene upregulation was shown by alizarin red staining on day7 and 14 post-delivery.

**Results:**

Proliferation of HOS cells treated with HA-NPs-APTES was similar to that of untreated cells. HA-NPs-APTES was visualized in cell cytoplasm within 24 hours. MiRNA-302a-3p level was upregulated in HOS, MG-63 and HmOBs as compared to untreated cells. As a result, COUP-TFII mRNA expression was reduced, followed by an increase of RUNX2 and other osteogenic genes mRNA expression. Calcium deposition induced by HA-NPs-APTES-miR-302a-3p in HmOBs was significantly higher than in untreated cells.

**Conclusion:**

HA-NPs-APTES may support the delivery of miRNA-302a-3p into bone cells, as assessed by osteogenic gene expression and differentiation improvement once this combination is used on osteoblast cultures.

## Introduction

The critical-sized bone defects after trauma, diseases, or surgical procedures are defects that cannot be completely healed by the physiological wound healing process. Thus, special surgical interventions mostly require bone tissue engineering to enhance healing [1]. Basically, bone tissue engineering principle consists in a triad composed of a bone scaffold, bioactive molecules and cells. The scaffold must be biocompatible and present physical and mechanical properties compatible with natural bone. Once placed in a defect, it will act as a framework for cells to adhere and differentiate to finally form new bone tissue [2]. The scaffold may be supplemented with bioactive molecules such as growth factors or oligonucleotides delivered by nanocarriers which play an essential role in promoting the cell proliferation and differentiation at the defect sites [2, 3]. Nanoparticles act as carriers for drugs and oligonucleotides to facilitate their cellular uptake. Several materials are being studied as nanocarriers such as Poly(DL-lactide-co-glycolide) (PLGA) [4], gold [5], SiO_2_ [6], and chitosan [7], they represent a very active and actual field of study [8].

MicroRNAs (miRNAs) are small, non-coding, single-stranded RNA which are currently used in a therapeutic approach for many diseases [9]. They function as post-transcriptional regulators leading to mRNA degradation after binding to the untranslated regions of mRNA [10]. The regulatory role of miRNAs has been reported in many biological processes [11] including bone formation [12]. Among the vast reservoir of the potentially bioactive molecules, miRNA-302a-3p was reported to stimulate murine osteoblast differentiation by blocking the repression exercised from COUP-TFII on RUNX2 expression [13]. Its overexpression is also active on osteoclastogenesis by regulating RANKL expression in human mandibular osteoblasts [14,15]. Beyond molecular effects, miRNAs suffer from poor biocompatible—and efficient methods of delivery. The challenges of delivering bare miRNAs include 1—poor cellular uptake of miRNA due to an overall-negative charge, 2—a short half-life under physiological conditions and 3—the triggering of a potentially unfavorable immune response [16]. The delivery system should stabilize small RNAs from degradation by nuclease enzyme and target them to the desired cell compartment within an optimal timing [17]. Cationic polyethyleneimine (PEI) or PLGA nanoparticles served for example as excellent carriers for miRNA but resulted in high cell cytotoxicity [18]. Lipid-based nanoparticles also efficiently deliver nucleic acid into the cell but may induce immune responses, and their short half-life strongly limits their usage [19].

Hydroxyapatite nanoparticles (HA-NPs) may represent an attractive solution [20, 21]. As they share common biochemical structures with natural bone, they present excellent biocompatibility and osteogenic properties. They bind efficiently to nucleotides via electrostatic forces between the positive charge of calcium ion and the negative charge of phosphate ions [22, 23]. Most of all, the nanoparticles are easily reproduced by common laboratory reagents and procedures. We demonstrated in a previous study that HA-NPs surface charge can be improved via a 3-aminopropyltriethoxysilane (APTES) chemical treatment. This modification resulted in a higher miRNA condensation and better HA-NPs uptake by osteoblasts [21]. By taking advantage of surface-modified HA-NPs properties, our purpose is to demonstrate that miRNA-302a-3p may be delivered in osteoblasts resulting in stimulation of osteoblast differentiation promptly in vitro.

## Materials and methods

### Hydroxyapatite modified with 3-aminopropyltriethoxysilane (APTES) nanoparticles synthesis

HA-NPs were synthesized and then surface modified by APTES as previously described [21]. Briefly, 4 mL oleic acid and 16 mL ethanol were added into 7.5 mL of the 0.25 M calcium nitrate solution containing 0.5 g polyethylene glycol (PEG6000) to prepare the mixture. The phosphate solution (0.15 M, 7.5 mL) was then dropped to the above mixture with agitation. The pH of the mixture was immediately adjusted to 10 with ammonia solution, then was hydrothermally treated at 120 °C for 10 h in a Teflon-lined autoclave, and finally water-cooled to room temperature. HA-NPs were collected by centrifugation, washed, and subsequently dried overnight. For surface modification with APTES, HA-NPs (0.2 g) were resuspended in 20 mL solution of APTES in anhydrous toluene (2.5% v/v) and stirred at room temperature for 3 h. Then, HA-NPs-APTES were collected by centrifugation, and washed with toluene to remove excess APTES. HA-NPs were dried at 60 °C for 24 h to produce the HA-NPs presented with terminal –NH_2_ groups.

To facilitate HA-NPs-APTES visualization after being internalized in HOS, a fluorescent tag (FitC) was added. HA-NPs-APTES (50 mg) were dispersed in 0.2 mg/mL of FITC (Merck KGaA, Darmstadt, Germany) in ethanol and stirred for 24 h. Then, FITC-tagged particles were centrifuged and washed with ethanol. HA-NPs-APTES-FITC were dried at 60 °C for 24 h.

### Cell culture

The study was approved by the ethics committee, Faculty of Dentistry, Chulalongkorn University (HREC-DCU 2022-038). Human mandibular primary osteoblasts (HmOBs) were collected and cultured as previously described [21]. The primary human osteoblasts from passage 5-8 were used in the following experiments. Human osteosarcoma cell lines, HOS (CRL-1543, ATCC) and MG63 (CRL-1427, ATCC), were cultured with Dulbecco’s Modified Eagle’s Medium with L-glutamine (DMEM; Gibco® by Life Technologies, NY, USA) supplement with 10% fetal bovine serum (FBS; Hyclone® Thermo scientific, Northumberland, UK), 1% antibiotic-antimycotic at 37 °C in a humidified atmosphere of 5% CO_2_. To prepare osteogenic medium, 0.05 mM ascorbic acid 2-phosphate, 100 nM dexamethasone, 10 mM b-glycerophosphate (Sigma-Aldrich) was added to the culture medium. Osteogenic medium was changed every 2-3 days.

### Conjugation of miRNA on HA-NPs-APTES and delivery to cells

HOS, MG63 or HmOBs cells (10^5^ cell/well) were cultured in 12-well plate for 24 hours. To prepare HA-NPs-APTES-miR, 50 µg/ml HA-NPs-APTES mixed with 5 nM miRNA-302a-3p (Qiagen, Hilden, Germany) in 10 µl RNase free water was dropped in 990 µl DMEM culture medium. Then, the suspension was added to the cultures. After 24 hours or 5 days, cells were harvested for RNA extraction. HA-NPS-APTES without miRNA were also added as control group.

### Resazurin assay

To evaluate the effect of bare nanoparticles on cell metabolic activity and proliferation, HOS cells (3000 cells/well) were cultured in 24-well plate for 24 hours. Then, HA-NPs-APTES (20, 50 and 100 µg/ml) in DMEM was added to the medium. Cells metabolic activity was assessed at day 4, 7, 11, 14 and 21 by using resazurin assay as described in previous study [24]. Briefly, 0.1 µg/ml resazurin (Sigma-Aldrich, MO, USA) in DMEM was added to the cultures for 4 hours before the supernatant was collected for OD measurement at 570 and 630 nm. The percentage of resazurin reduction was then calculated.

### Fluorescent microscopy imaging

HOS cells (10^4^ cell/well) were cultured in 8-well culture slide (ibidi, Germany, Cat.No:80826) for 24 hour to reach 80% confluency. Then, FitC tagged HA-NPs-APTES (50 µg/ml) were added and co-cultured for the next 24 hours. Then, cells were washed and stained by 5 µg/ml FM® 4-64 lipophilic styryl dye (Thermo Fisher scientific, MA, USA) on ice for 5 minutes, and fixed with 4% formaldehyde for 10 minutes on ice. Cells were washed 3 times with Hanks’ balanced salt solution (HBSS; Invitrogen, CA, USA, Cat.No:14175079). Nikon A1r (Nikon, Tokyo, Japan) Spectral confocal microscope was used to visualize nanoparticle inside cells by capturing Z-stack images with 50 µm distance between each image. The 3D-constructed images and animation were processed by Imaris 9.5 (Oxford instruments) software.

### Scanning electron microscopy (SEM)

Scanning electron microscopy (Sigma 300 VP, Zeiss, Oberkochen, Germany) was used to visualize morphology of HOS cells treated with HA-NPs-APTES. HOS cells (1 × 10^4^/well) were cultured with 50 µg/ml HA-NPs-APTES for 24 hours. Before imaging, samples were fixed in 4% formaldehyde for 10 min then rinsed with 0.9% normal saline and dehydrated with serial concentration of ethanol from 60 to 100% then coated with a 20 µm thick layer of gold.

### Reverse transcription and quantitative polymerase chain reaction

Total RNA was extracted by Trizol lysis reagent (Invitrogen, CA, USA,) according to the manufacturer protocol. One microgram of total RNA was converted to cDNA by miScript II RT Kit (Qiagen, Hilden, Germany) on a thermal cycler (LifePro, Bioer, Hangzhou, China). For detection of miRNA, quantitative PCR was performed using Quantitect SYBR Green PCR Master mix (Qiagen, Hilden, Germany) on PCR detection system (StepOnePlus, Applied Biosystem, CA, USA). The sequences of miRNA-302a-3p and RNU6-2 primers are shown in Table 1. The PCR condition was 95 °C for 15 min followed by 40 cycles of amplification consisting of 94 °C for 15 sec, 55 °C for 30 sec, and 70 °C for 30 sec. The expression level of miRNA was normalized to miScript PCR controls RNU6-2.

**Table 1:** Primer sequence used in the study.

Primer	Sequence	
	Forward	Reverse
miRNA-302a-3p	UAAGUGCUUCCAUGUUUUGGUGA	N/A
RNU6-2	GTGCTCGCTTCGGCAGCACA	N/A
COUP-TFII	CAAGGCCATAGTCCTGTTCACC	CGTACTCTTCCAAAGCACACTGG
RUNX2	CCGGAATGCCTCTGCTGTTATGA	ACTGAGGCGGTCAGAGAACAAACT
ALP	CGAGATACAAGCACTCCCACTTC	CTGTTCAGCTCGTACTGCATGTC
OCN	CTAGAGCGGGCCGTAGAAGCG	ATGAGAGCCCTCACACTCCTC
OSX	CGGGACTCAACAACTCT	CCATAGGGGTGTGTCAT
GAPDH	TCATGGGTGTGAACCATGAGA	GCTAAGCAGTTGGTGGTGCA

For detection of other genes, quantitative PCR was performed using a SensiFAST™ Kit (Meridian bioscience, MI, Italy, Cat.no BIO-82005) on PCR detection system (StepOnePlus, Applied Biosystem, CA, USA). Primer sequences for GAPDH, COUP-TFII, RUNX2, ALP, OCN, and OSX are shown in Table 1. The PCR condition was 95 °C for 2 min, followed by 40 amplification cycles consisting of 95 °C for 15 seconds, 60 °C for 30 seconds. Reactions were performed in duplicate, and averages were used for analysis. GAPDH expression was used as an internal control. Fold expressions were calculated by 2^-ΔΔCT^ method.

### Alizarin red staining of calcium deposition

HmOBs (10^5^ cells/well) were cultured in 6-well plate overnight, then HA-NPs-APTES or HA-NPs-APTES-miR were added in culture media. For osteogenic media group, after cells seeding overnight the culture media was replaced with osteogenic media along with HA-NPs-APTES or HA-NPs-APTES-miR. The nanoparticles were given only one time. The media was replaced every 2-3 days. After 7 or 14 days, the supernatant was removed and HmOBs were washed with PBS 2 times and then fixed with cold methanol for 10 min. After fixing, cells were washed with deionized water then stain with Alizarin red stain for 2 min. After staining, cells were washed with deionized water and dried in room air.

Alizarin red was quantified by adding 20% methanol and 10% acetic acid in the stained plate. After 15 minutes, liquid is transferred to measure optical density at a wavelength of 405 nm.

### Statistical analysis

The data were expressed as the mean values ± standard deviation. Data normalities were tested, and statistical analyses were performed by one way analysis of variance and Tukey post hoc or Student’s *t*-test by SPSS v 21.0 statistical software package. The differences were considered statistically significant when *p* value is equal or less than 0.05.

## Results

### Proliferation of HOS cells after HA-NPs-APTES treatment

**Fig. 1:**
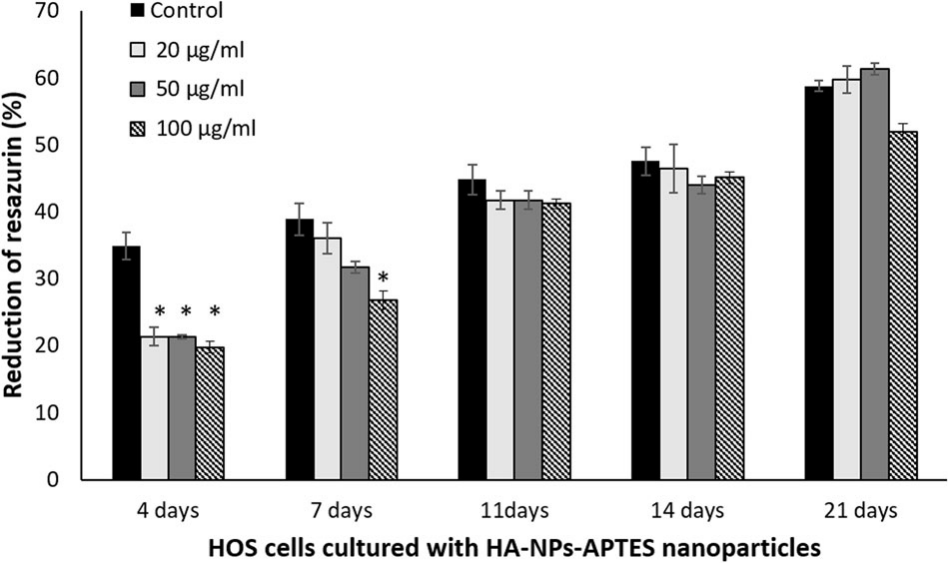
Resazurin reduction of HOS after treatment with HA-NPs-APTES. Three concentrations (20, 50 and 100 µg/ml) of HA-NPs-APTES were added to culture medium. Resazurin assay were done at day 4, 7, 11, 14 and 21. Graphs represent means from 3 replicates with standard deviation. Student’s *t*-test demonstrated significant differences of resazurin reduction in treated cells when compared to the control. *p ≤ 0.05.

The percentage of resazurin reduction, reflecting the number of living cells, gradually increased from day 4 to day 21 in all concentrations of HA-NPs-APTES (Fig. 1).

After 4 days of culture, decrease in resazurin reduction was observed with all HA-NPs-APTES concentrations when compared to untreated control (20 µg/ml = 21.34 ± 0.46%, 50 µg/ml = 21.32 ± 0.69%, 100 µg/ml = 19.76 ± 0.88%, control = 34.91 ± 7.97%). This difference remained visible only for 100 µg/ml HA-NPs-ATPES at day 7 (26.8 ± 1.52%, control = 38.89 ± 2.42%) and the whole conditions behave the same until day 21.

The highest HA-NPs-APTES concentration with the lower effect on cell proliferation on day 7 was chosen to be tested for intracellular uptake and osteogenic differentiation. Therefore, 50 µg/ml HA-NPs-APTES were used in the following experiments for the rest of the study.

### Visualization of HA-NPs-APTES uptake by HOS

**Fig. 2:**
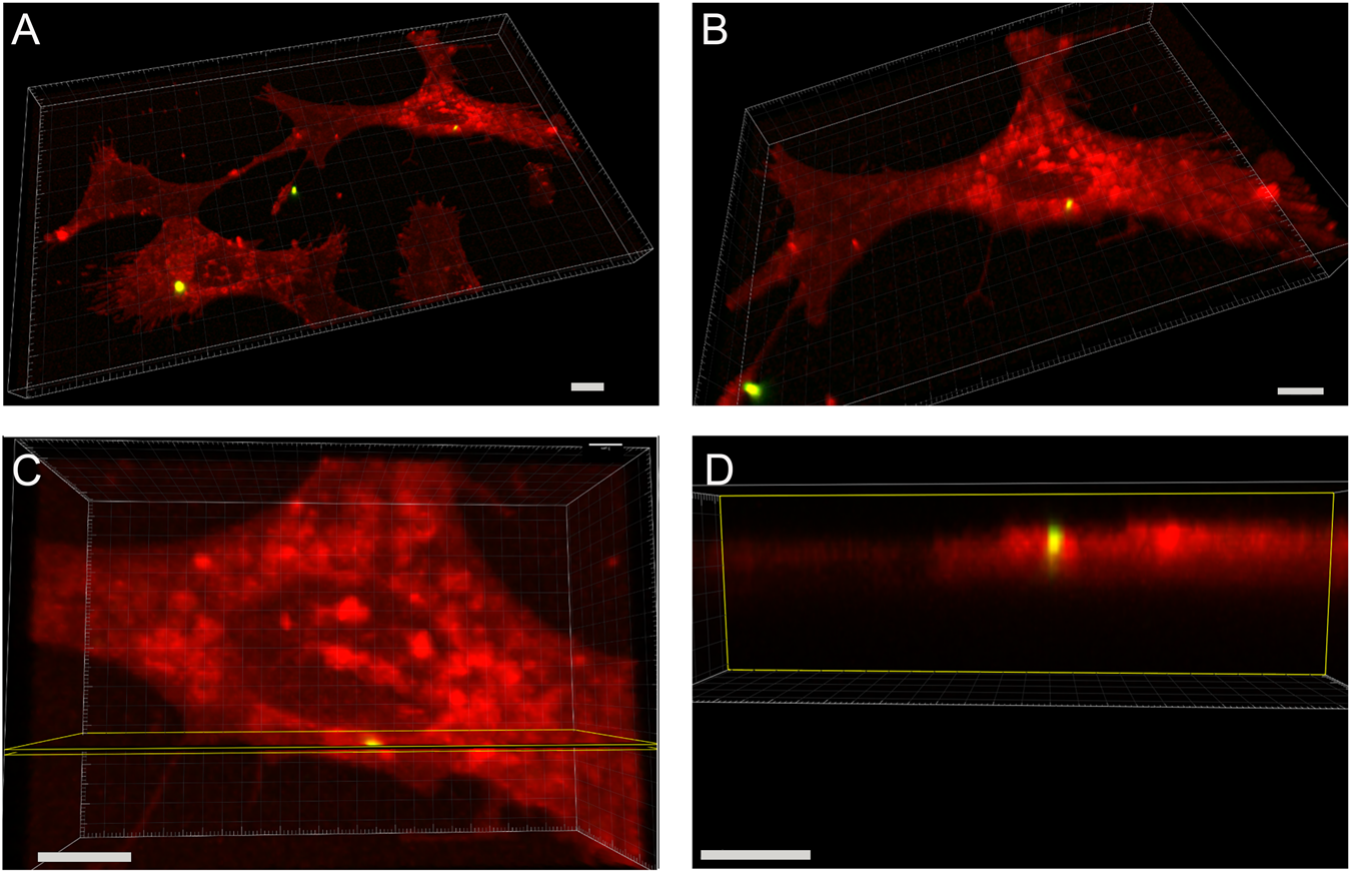
3D construct image of HA-NPs-APTES uptake by HOS cells. Representative area from culture plate is selected. Serial Z-stack images were captured with range of 50 µm between each image. **A** 3D model from Z-stack images, **B** 3D model showed single cell, **C** Selected area of vertical section, **D** Vertical section of area selected in **C**. Scale bar = 5 µm.

**Fig. 3:**
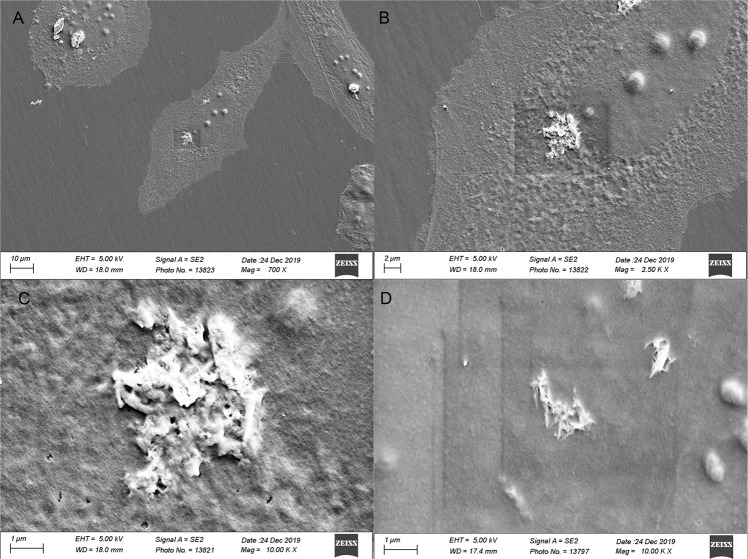
SEM imaging of HA-NPs-APTES uptake by HOS cells. Morphology of cells after treatment with HA-NPs-APTES for 24 hours. **A** 700× magnification showing multiple cells with HA-NPs-APTES. **B** 2500× magnification showing HA-NPs-APTES in one cell. **C**, **D** 10,000× magnification showing rod-shaped nanoparticles inside the cell. The dark squares seen in the figure were artifacts due to high voltage, the images were not modified.

After 24 hours of culture with HA-NPs-APTES-FitC, cells were visualized by confocal microscopy. HA-NPs-APTES internalization was confirmed by serial Z-stack images (Fig. 2, Supplementary video 1-3). Particles were localized in cytoplasm compartment outside the nucleus; however, it was not possible to discriminate whether they were contained into organelles or in the cytoplasm. Internalization and localization in close vicinity to nuclei were confirmed in SEM images. Membrane integrity was preserved above HA-NPs-APTES (Fig. 3).

### Delivery of miRNA302a-3p by HA-NPs-APTES and its effect on osteogenic differentiation

#### Effects on gene expression

To demonstrate that HA-NPs-APTES can deliver functional miRNA, miRNA 302a-3p conjugated to HA-NP-APTES (HA-NPs-APTES-miR) were suspended in the culture medium of the osteosarcoma cells, HOS and MG63, and the primary human mandibular osteoblasts (HmOBs). After 1 and 5 days of culture, the expression of miRNA 302a-3p and mRNAs including COUP-TFII, RUNX2, ALP, OCN and OSX were assessed by qPCR. Cells in contact with HA-NPs-APTES alone-without miR were added for a control condition.

**Fig. 4:**
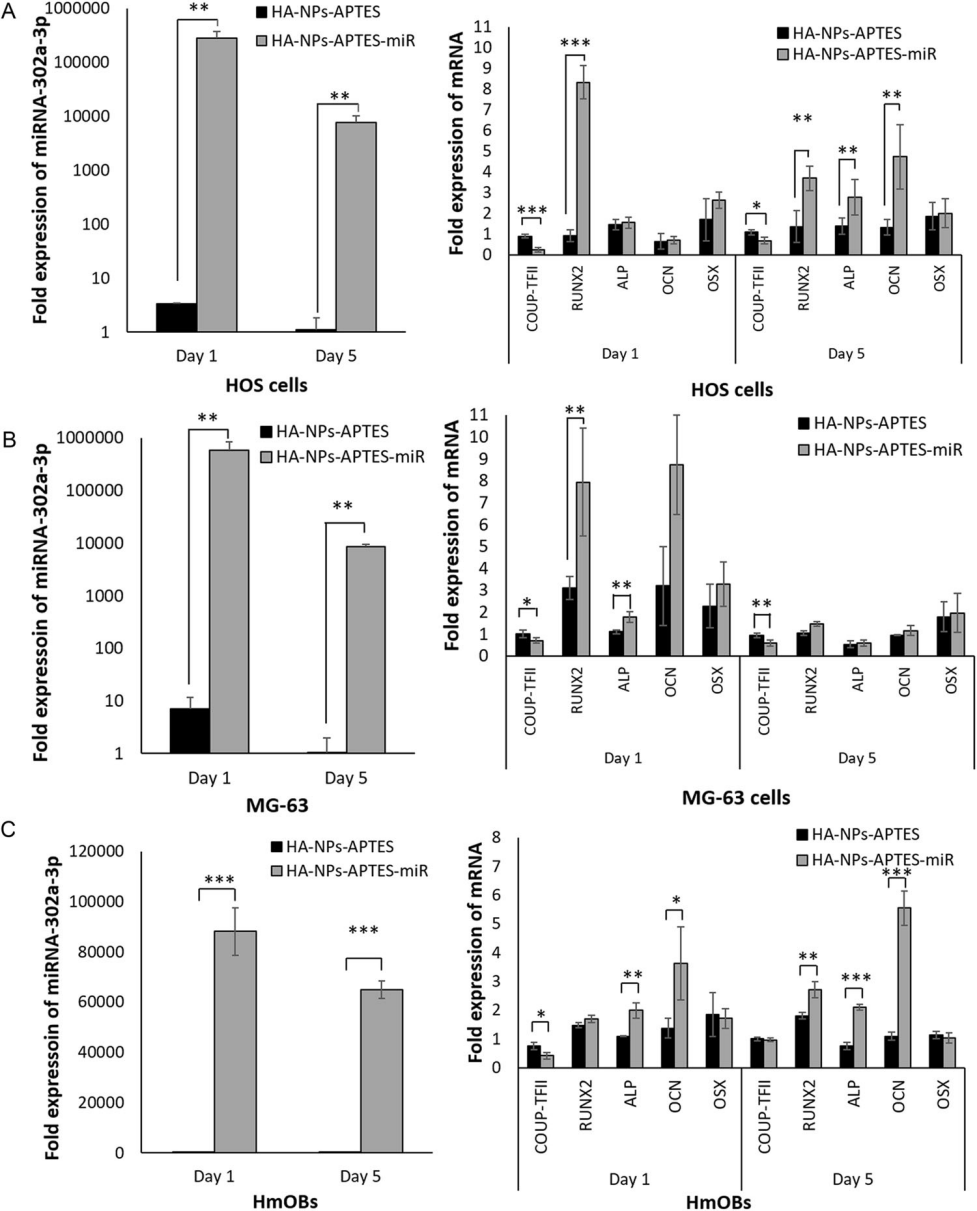
Delivery of functional miRNA 302a-3p by HA-NPs-APTES. Expression of miRNA-302a-3p, COUP-TFII and osteogenic genes in HOS MG63 cell lines and HmOBs were measured by qPCR. **A** HOS cells at 1 and 5 days of treatment with HA-NPs-APTES-miRNA. **B** MG63 cells at 1 and 5 days of treatment with HA-NPs-APTES-miRNA. **C** HmOBs at 1 and 5 days of treatment with HA-NPs-APTES-miRNA. Student’s *t*-test demonstrated significant differences in miRNA and mRNA expression in HA-NPs-APTES-miR treated cells compared to HA-NPs-APTES treated cells at the same date. Expressions of miRNA were normalized to the RNU6-2 gene. Expressions of mRNA were normalized to GAPDH gene. *p ≤ 0.05, **p < 0.01, ***p < 0.001.

On day 1, the expression of miRNA 302a-3p was significantly increased to 2.81 × 10^5^ ± 0.90 × 10^5^ folds in HOS and 5.84 × 10^5^ ± 2.76 × 10^5^ folds in MG63, higher than the baseline control receiving HA-NPs-APTES alone-without miR (Fig. 4A, B). On day 5, miRNA 302a-3p expression was slightly lower than day 1, however the relative expression remained significantly higher than the baseline control (Fig. 4A, B). In HmOBs, miRNA 302a-3p expression was increased to 8.81 × 10^4^ ± 0.95 × 10^4^ folds on day 1, and to 6.49 × 10^4^ ± 0.3 × 10^4^ folds on day 5 (Fig. 4C).

An increase of miRNA 302a-3p in HOS, MG63 and HmOBs resulted in a modulation of the target gene expression. Those included down regulation of COUP-TFII, up-regulation of RUNX2, ALP and OCN. By contrast, OSX expression was not modified. The effect of miRNA 302a-3p on the target genes were more pronounced on day 5 in HOS and day 1 in MG63 but remained stable over time in HmOBs (Fig. 4A–C).

#### Effects on cell differentiation—mineralization

**Fig. 5:**
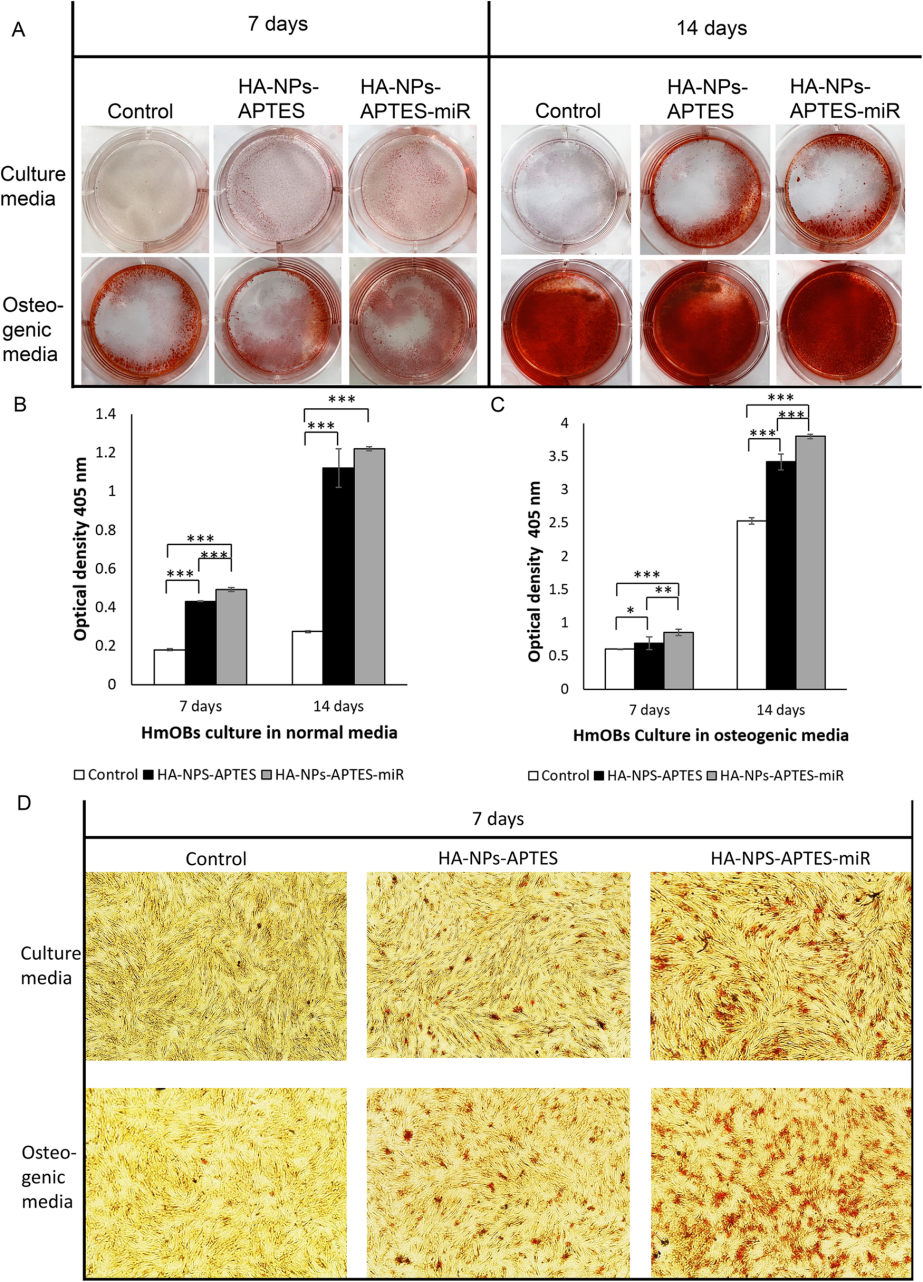
Alizarin red staining of HmOBs treated with HA-NPs-APTES or HA-NPs-APTES-miR. **A** HmOBs were cultured with HA-NPs-APTES or HA-NPs-APTES-miR in normal culture medium or osteogenic medium. The culture plates were examined and photographed on day7 and day14. The quantity of alizarin red staining of HmOBs cultured (**B**) in normal media, or (**C**) osteogenic media was measured on the spectrophotometer at a wavelength of 405 nm. **D** Normal cell morphology and calcium deposition showing in red area of day7 cultures were observed through 40× magnification. Photographs of cells without treatment, or with HA-NPs-APTES, demonstrated calcium deposition area much smaller than cells with HA-NPs-APTES-miR. *p ≤ 0.05, **p < 0.01, ***p < 0.001.

Calcium deposition induced by osteogenic gene expression was then visualized in HmOBs treated with HA-NPs-APTES and HA-NPs-APTES-miR by using alizarin red staining (Fig. 5A). Effects of miRNA 302a-3p were assessed in normal culture medium, or in osteogenic medium.

In normal medium (Fig. 5B), HmOBs showed a detectable level of mineralization that slightly increased from day 7 to 14. Upon stimulation with osteogenic medium (Fig. 5C), this expression was improved by 2 and 5 folds on day 7 and 14, respectively. By contrast, HA-NPs-APTES induced higher cell mineralization in normal culture medium which increased from day 7- 14. In osteogenic medium, an additional effect on cell mineralization was induced by the HA-NPs-APTES alone, either on 7 or 14 days.

Increase of miRNA 302a-3p by adding HA-NPs-APTES-miR, however, significantly improved cell mineralization induced by HA-NPs-APTES alone, by approximately 20% on day 7 and 14, either in normal or osteogenic medium. Whichever treatment applied on HmOBs, cell morphology remained normal, depicting a confluent layer of fusiform cells. Once cells were stained with alizarin red, calcium deposition could be easily observed (Fig. 5D). HmOBs proliferation rate of cells with HA-NPs-APTES and HA-NPs-APTES-miR remained consistent with the control cells without any treatment.

## Discussion

In the present report, we conducted experiments to show that HA-NPs-APTES were biocompatible to be internalized by osteoblastic cells and therefore HA-NPs-APTES can serve as a carrier for miRNA delivery. After internalized, the miRNA-302a-3p was overexpressed and regulated osteogenic gene expression.

The biocompatibility of the HA-NPs-APTES was assessed in HOS osteoblastic cells cultured with multiple concentrations of the nanoparticles. A delay in HOS proliferation was observed during the initial stages, up to 4 days in culture. This delay was however surpassed after 7 days, except for the highest concentration (100 µg/ml) that needed 3 more days for a complete restoration to normal cell proliferation. The high concentration of hydroxyapatite nanoparticles at 1 mg/ml are known to reduce viability of osteoblast cells [25], which is consistent with our results. Likewise, other study found that high concentrations of calcium ion coming from HA-NPs dissolution could decrease proliferation of mesenchymal stem cells [26]. Most of the HA-NPs-APTES in our conditions were dissolved after 7 days in culture. Even if we did not measure Ca ions concentrations, we assume that it was largely decreased from day 7 and that it may explain the concomitant proliferation recovery in treated cells. According to cell biocompatibility, this study was conducted with the highest nanoparticle concentration at 50 µg/ml that did not affect cell proliferation on day 7. Since our previous study demonstrated that the maximal nanoparticles concentration allowed a maximal condensation of miRNA^21^, we assumed that this concentration would be the most efficient in terms of miRNA delivery as well as of cell proliferation and differentiation.

The HA-NPs-APTES-miR were internalized in cell cytoplasm, as shown by confocal microscopy and SEM images. MiRNA-302a-3p was then highly expressed inside the cells to function on its targets, as demonstrated by reduction of COUP-TFII mRNA expression and osteoblastic gene upregulation. As the mechanism of delivery, we hypothesized that miRNA and HA-NPs-APTES escaped the endosomal pathway by the proton sponge effect [27]. Indeed, cationic nanoparticles like HA-NPs-APTES are dissolved in acidic endosomes upon endocytosis. Increasing Ca concentration inside these particles creates an osmotic current of fluids inside these particles whose membranes eventually rupture to release their content inside the cytoplasm [28].

With the aim to create bone substitutes with osteoinductive properties, we used the miRNA-302a-3p, a potent activator of osteogenic differentiation of mouse osteoblasts [13] and that is released naturally in exosomes from mature-differentiated osteoblasts to induce mouse bone marrow-derived stromal cell line, ST2 [29]. We assumed that miR-302a-3p would also activate human cells differentiation since the binding sites of the 3’-UTR of COUP-TFII mRNA, the specific target of miRNA-302a-3p, are conserved among species including human [13]. To our knowledge, miRNA-302a-3p effects on human cells have never been described before.

The effect of miRNA-302a-3p on reducing COUP-TFII mRNA level was equivalent in all cell types demonstrated in this study. It was also effective on osteogenic gene upregulation, but with a time shift of about 5 days depending on the cell line. Upregulation of the osteogenic profile was mostly observed after 1 day in MG63 and after 5 days in HOS. Increase of nanoparticles uptake was shown during cell proliferation [30]. MG63 are known as a fast-proliferative cell line when compared to HOS [31]. Thus, the observed 5-day shift may be explained by the discrepancy in proliferation rates of these 2 cell types. Furthermore, MG63 is a pre-osteoblastic cell line which is unable to differentiate into mature osteoblast [30]. Effects on gene expression observed from day 1 may therefore have led to an optimal differentiation level thanks to i. the miRNA and ii. the culture conditions. The related transcriptional upregulations may therefore have been reset.

HmOBs are primary cells and therefore harbor proliferative, transcriptomic, and metabolic characteristics closer to bone cells in vivo. In general, these primary cells proliferate slower and are more resistant to nucleic acid transfection than cell lines [32]. This is probably the reason why miRNA-302a-3p expression level in HmOBs was lower than in HOS and MG63. In addition, the miRNA-302a-3p expression was absolutely the result of HA-NPs-APTES-miR delivery, as the control cells only showed low constitutive expression level at the same condition. Although the level in primary bone cells was lower than in the bone cell lines, the delivered miRNA-302a-3p in HmOBs was sufficient to stably regulate osteogenic genes over time from day 1 to 5. Finally, the decrease of efficacy of miRNA-302a-3p on COUP-TFII mRNA expression at day 5 may be due to its decreasing expression level. Even though the expression of miRNA is still high at day 5, It may not totally escape endosomal pathway to reach an effective amount in cytoplasm. A study by Gilleron et al. 2013 thus demonstrated that the amount of miRNA delivered into cells may not be proportional to its function and that only 1–2% of siRNA carried by lipid nanoparticles escape the endosomal compartments to function in cytosol [33].

Osterix is a key transcription factor involved in late maturation of bone cells, especially for mineralization [34]. In this study, HA-NPs-APTES upregulated osterix, but an addition of miRNA-302a-3p did not significantly change the osterix mRNA level in all cell types. Nonetheless, HA-NPs-APTES alone sufficiently activated high level of mineralization in HmOBs, even in cells cultured without conditioned medium. Therefore, we postulated that HA-NPs-APTES might stimulate osterix expression to its saturated level so that additional effect via miRNA-302a-3p activity could not be observed. A beneficial effect of miRNA-302a-3p on mineralization in HmOBs may be resulted from the function of other osteogenic genes, especially in earlier phases of bone cells differentiation.

## Conclusion

MicroRNA can be delivered efficiently by using HA-NPs-APTES as a carrier. After being internalized, miRNA-302a-3p overexpression increases human mandibular osteoblast cells differentiation through the regulation of osteogenic genes and enhances bone mineralization. The nanoparticles therefore serve as a carrier to deliver miRNAs as efficient bioactive molecules for bone or periodontal tissue regeneration.

## Supplementary information

Supplement video 1

Supplement video 2

Supplement video 3

Supplement video legends
