# Supplementary material for: Delivery of microRNA-302a-3p by APTES modified hydroxyapatite nanoparticles to promote osteogenic differentiation in vitro
Source: BDJ Open. 2023 Feb 22;9:8. doi: 10.1038/s41405-023-00135-x (PMC9947005; doi:10.1038/s41405-023-00135-x)
Supplement: Supplementary file 4 — Supplement video legends [file 41405_2023_135_MOESM4_ESM.docx]

**SI Video 1**

HA-NPs-APTES within single cell. HA-NPs-APTES (yellow) was shown close to the nucleus of HOS cell

**SI Video 2**

Higher magnification of HA-NPs-APTES within the cell. HA-NPs-APTES adjacent to the nucleus of HOS cell from supplement video 1 was demonstrated at higher magnification

**SI Video 3**

Cross section serial image of HA-NPs-APTES inside the cell. Serial image of compartment in supplement video 2 showing HA-NPs-APTES inside cell membrane area.
